# The reproducibility gap in graph neural network workflows for cell dynamics: A checklist‐driven case study

**DOI:** 10.1111/jmi.70139

**Published:** 2026-06-23

**Authors:** Martin Schätz, Ko Sugawara

**Affiliations:** ^1^ Department of Mathematics Informatics and Cybernetics University of Chemistry and Technology Prague Czech Republic; ^2^ Laboratory for Developmental Dynamics RIKEN Center for Biosystems Dynamics Research Kobe Japan

**Keywords:** bioimage analysis standards, computational reproducibility, graph neural networks, quantitative microscopy

## Abstract

As part of a Global BioImage Analysts' Society (GloBIAS) initiative, we evaluated the reproducibility of a Graph Neural Network (GNN) study on cell dynamics using structured, community‐developed checklists from the Quality Assessment and Reproducibility for Instruments and Images in Light Microscopy (QUAREP‐LiMi) initiative. Notably, these checklists were published after the 2022 Target Paper, meaning our assessment is necessarily retrospective. Our assessment revealed a gap between recent reporting standards and practical execution. Reproduction attempts across multiple environments confirmed deficiencies, including the absence of explicit image metadata (pixel size and timestamps), which limits quantitative interpretation. Furthermore, the absence of a software container, which was typical for the time of publication and the incomplete dependency list, necessitated complex manual environment configuration, increasing the barrier to entry. This experience highlights the contrast between the reproducibility expectations at the time and newer standards achieved by minimal compliance and true functional access. We conclude by presenting a detailed discussion of the broader implications for the quantitative biology community and propose actionable recommendations including robust environment containerisation and standardised data deposition to ensure complex computational workflows become scientifically sound and reusable resources. This retrospective case study evaluates a legacy computational paper against modern reproducibility framework, not to critique past non‐compliance, but to extract actionable lessons for future research standards

## 1 INTRODUCTION

As part of the Global BioImage Analysts' Society^1^ (GloBIAS) initiative,^1^ this paper presents a structured, checklist‐driven retrospective evaluation of the practical reproducibility of the image‐analysis and Graph Neural Network (GNN) workflow described in the study ‘Probing the rules of cell coordination in live tissues by interpretable machine learning based on graph neural networks'^2^ (the Target Paper). This assessment is intentionally retrospective. The Target Paper was published in September 2022, meaning it is approximately four years old at the time of this analysis (2026). This study is part of a bigger initiative involving seven teams testing the reproducibility of several papers.

Graph Neural Networks have emerged as powerful tools for modelling multicellular dynamics, but they also represent workflows with unusually high reproducibility demands: complex software environments, multi‐step processing pipelines, and strong dependence on metadata completeness. Revisiting a prominent GNN‐based biological study therefore provides an opportunity to understand how well such workflows survive the test of time and how evolving reproducibility standards reveal new strengths and weaknesses.

In this work, we apply the QUAREP‐LiMi community‐developed checklists^3^ to evaluate the published code, data, documentation quality, metadata completeness, and environment transparency of the Target Paper. Our primary goal is to map the gap between stated intentions for reproducibility and the practical, user‐facing reality of executing the workflow. Our work aligns with a broader movement toward improving computational reproducibility across scientific domains. For example, the REFORMS Consortium recently published consensus‐based recommendations for machine‐learning–based science (Science Advances, 2024), reflecting a cross‐domain initiative similar in spirit to the QUAREP‐LiMi and GloBIAS efforts.

Crucially, the QUAREP‐LiMi reporting checklists used in our evaluation—covering Image Data, Machine Learning workflows, and computational Workflow transparency—had *not yet been officially published* at the time of the Target Paper's release. Although the corresponding preprint was publicly available on 14 February 2023, the finalised versions of these community standards appeared later in 2023.^3^

Therefore, the purpose of this study is not to fault the Target Paper for non‐compliance with standards that did not yet exist. Rather, our aim is to use modern, community‐developed reproducibility frameworks to illuminate how earlier computational studies fare when viewed through today's expectations, and to identify actionable lessons for the future.

By situating this evaluation within the GloBIAS initiative and by explicitly acknowledging its retrospective nature, we aim to highlight broader lessons for the community: how reproducibility expectations have matured, how historical workflows can be assessed constructively using modern tools, and how to accelerate progress toward robust, transparent, and verifiable computational biology pipelines.

### 1.1 Target Paper overview

The Target Paper, ‘Probing the rules of cell coordination in live tissues by interpretable machine learning based on graph neural networks'^2^ introduces a novel analytical framework for understanding cell fate determination in tissues.

#### 1.1.1 Motivation

The stability and robustness of developing and homeostatic tissues depend on various types of spatiotemporal cell‐to‐cell interactions. Although live imaging and cell tracking offer direct evidence of cell coordination rules, extracting and comparing these rules across diverse tissues—which may operate on different length and timescales of coordination—requires a versatile analytical framework. The research aims to infer cell‐to‐cell interaction rules from live images of multicellular dynamics, thereby understanding how cell coordination shapes complex developmental outcomes, notably cellular lineage commitment. Existing analyses of skin stem cells, such as those conducted on the mouse hind paw epidermis, were often hypothesis‐based and might fail to extract suppressive effects. Therefore, a generic framework capable of suggesting coordination rules in an unbiased manner was necessary.

#### 1.1.2 Approach

The authors propose using Graph Neural Network (GNN) models because they are well‐suited for predicting cell fate and inferring the underlying cell interactions that govern multicellular dynamics.

1. Data graph construction: Live‐image data and cell tracks (including divisions and delaminations) from the basal layer of the epidermis are converted into spatiotemporal graphs. Nodes represent cells in a specific time frame, and edges encode spatial relationships (cell‐to‐cell contact) and temporal relationships (lineage tracks).
2. Model structure: The GNN model, termed the bidirectional spatiotemporal GNN model, processes information through sequential message passing (MP): backward temporal MP (future to past), spatial MP (bidirectionally on contacts), and forward temporal MP (past to future). Here, the GNN model is trained as a three‐class classification problem to predict future cell fates—division (Div), delamination (Del), or no behaviour (NB)—from past observations, and therefore does not perform retrospective inference. The backward temporal MP (future to past) operates solely within the input graph and does not involve access to future information or introduce information leakage. The model was applied using datasets of live mammalian epidermis (hind paw^4^ and ear skin stem cells^5^).
3. Interpretability: To extract the rules learned by the machine, the researchers employed the attribution method called Integrated Gradients (IG), which quantifies the impact of each input variable on the output prediction score.

#### 1.1.3 Solution

The GNN framework (Figure 1) proved successful in inferring cell interaction rules without prior knowledge of the signalling mechanisms involved. In this study, the Area Under the Curve (AUC), specifically the ROC‐AUC obtained from the Receiver Operating Characteristic (ROC) curve, was used as the primary evaluation metric to assess classification performance. In classification tasks, ROC‐AUC plots the true positive rate against the false‐positive rate across different decision thresholds; an ROC‐AUC of 0.5 corresponds to random guessing, while higher values indicate better discriminative ability.

- Prediction success: The GNN model achieved AUC scores significantly higher than random prediction (AUC = 0.5) for all cell behaviours (Div, Del, NB), demonstrating meaningful predictive capability. The highest performance was obtained when both cell area and Fucci‐G1 signal were included as input features, yielding AUC values of approximately 0.9 for Div, 0.75 for NB, and 0.6 for Del, indicating that Div is the most predictable class. When these features were removed, the performance decreased substantially, confirming their importance for accurate prediction; nevertheless, the model still achieved performance higher than random prediction even without node features, suggesting that the graph structure itself encodes useful information for predicting cell fate.
- Discovery of rules: The analysis discovered distinct neighbour cell fate coordination rules between different regions of the body (hind paw vs. ear epidermis).
- In the hind paw, the model confirmed the previously reported effect of Delamination‐induced neighbouring Division (with a delayed effect of 1–2 days). It also suggested a potentially novel mechanism where neighbouring Delamination suppresses Delamination in the next frame (lateral inhibition).
- In the ear skin data, an additional coordination rule was found, suggesting a mechanism of neighbouring Division‐induced Delamination and Division‐suppression, which was absent in the hind paw epidermis.
- Validation: By applying the attribution method to numerical simulations, the authors confirmed that the GNN accurately extracted the artificially implemented coordination rules (e.g., Delamination‐induced Division and Division‐induced Delamination).

**FIGURE 1:**
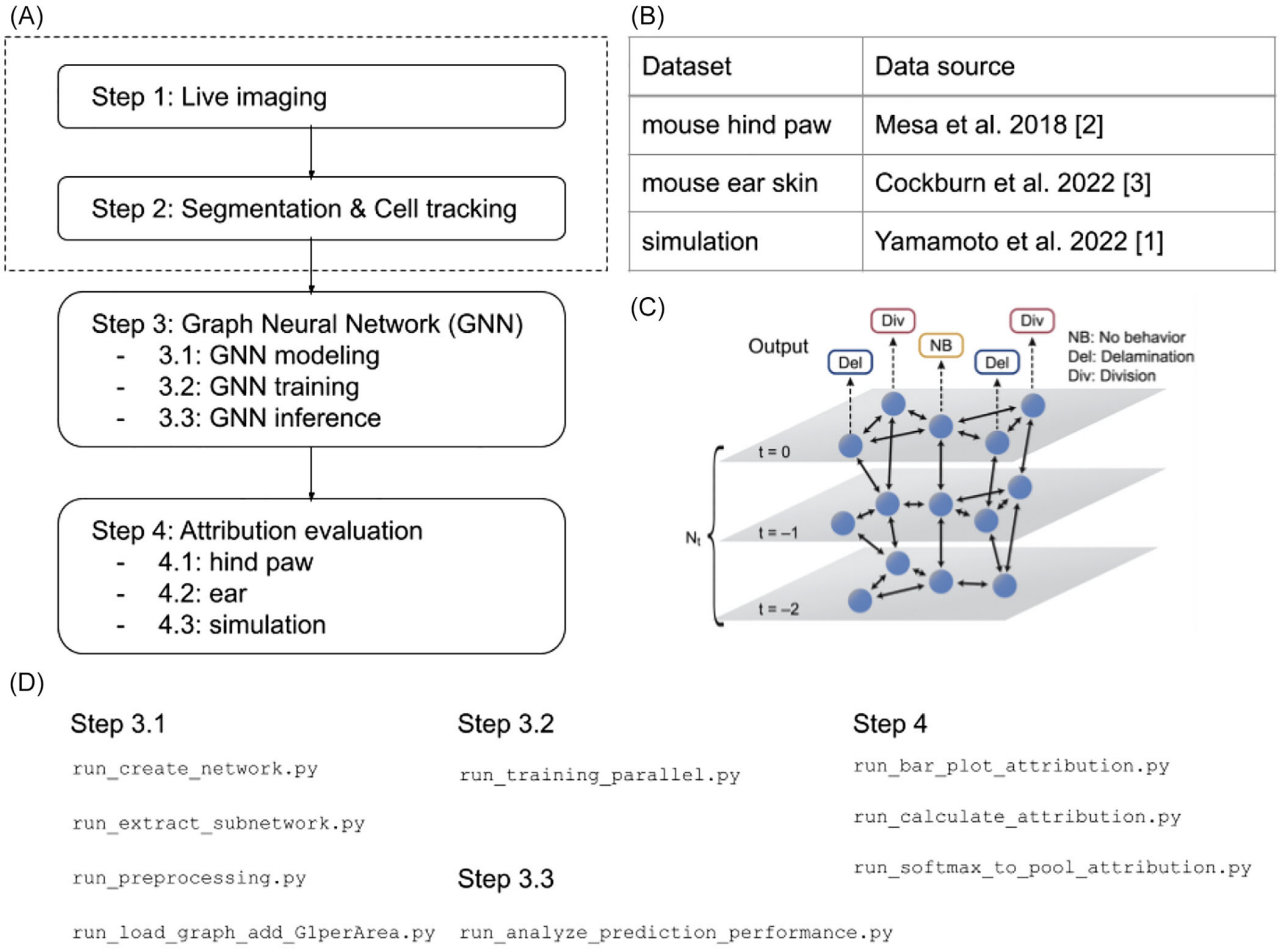
Simplified workflow overview. (a) Overall workflow steps of the used approach in the target paper. Steps 1 and 2 were not in the scope of the target paper. It was part of previous research. (b) Source data overview.^2^, ^4^, ^5^ (c) Figure adapted from [2] licensed under CC‐BY 4.0. (d) Overview of the subsequent functions that must be executed, in the required order, to complete workflow steps 3 and 4.

#### 1.1.4 Aims for reusing the results

The authors aimed to facilitate the reuse and extension of their results by providing resources and proposing broad applicability for the framework:

- Data and code availability: The paper states that the pipeline of the GNN framework and the analysed data (tracks of cells in the epidermis and numerical simulations) are available on a GitHub repository.
- Broad applicability: The GNN framework is considered applicable not only to the current epidermis data but also for general multicellular dynamics, such as those in developing embryos, malignant tissues, and organoids.
- Applicability beyond biology: The method is useful for a wide range of systems where modelling by stochastic interacting agents is effective, including disease spreading and ecology.
- Methodological extension: The attribution methods employed are intended to lay the groundwork for developing methods capable of interpreting the effects of higher‐order subgraph motifs (e.g., three‐body cell interactions or fate imbalance across generations), potentially using techniques like attention‐based GNN models.

## 2 METHODOLOGY

Our evaluation is based on a standardised, multi‐pronged protocol designed to test the full spectrum of reproducibility, from data reporting to code execution.

### 2.1 Applied checklists and standards

The assessment was guided by three complementary, community‐developed checklists,^3^, ^6^ ensuring a comprehensive critique tailored to the Target Paper's specific nature (quantitative imaging and machine learning). These checklists, created by the ‘Image Analysis and Visualisation Working Group’ within the Quality Assessment and Reproducibility for Instruments and Images in Light Microscopy (QUAREP‐LiMi) initiative, are designed to enhance the clarity and reproducibility of light microscopy images and their analyses in publications.^2^ Our approach consists of a systematic evaluation of the selected published paper and data against the three defined reporting levels: ‘minimal’ (necessary requirements), ‘recommended’ (measures for understandability), and ‘ideal’ (future considerations). This practical application of the checklists will allow for a comprehensive evaluation of how well published image figures and image‐analysis workflows adhere to established standards for reproducibility. The following checklists will be used:

1. **Checklist for image publication**:^6^ This checklist provides key recommendations for preparing light microscopy images for publication to enhance their clarity and interpretability. It covers critical aspects such as Image Format (e.g., appropriate cropping, rotation, separation of individual images, showing example images for quantification and phenotype ranges), Image Colours and Channels (e.g., clear annotation of channels, proper brightness/contrast adjustment, high‐visibility colours, provision of greyscale versions, and intensity scales), Image Annotation (e.g., adding legible scale information, explaining all annotations, and including important imaging details), and Image Availability (e.g., sharing images, preferably lossless compressed or original files, and making them freely downloadable via public or dedicated image databases).
2. **Checklist for publication of image‐analysis workflows** ^6^: These checklists offer structured guidelines for transparently documenting image‐processing and analysis steps, which are essential for the reproducibility of results derived from image analysis. They are further divided into three specific categories based on the nature of the workflow:

- Established workflows: Focuses on documenting workflows already available in scientific literature or well‐established within specific fields, requiring citation of the workflow and platform, reporting of key settings, provision of example input/output data, and documentation of any manual interventions (e.g., regions of interest), and precise version numbers of the workflow and software.
- New workflows: Addresses novel sequences of established or new image‐analysis components, necessitating detailed description and citation of individual components, specification of exact software versions, description of the application sequence, documentation of key settings, and sharing of the developed workflow as code or pipelines along with example data.
- Machine learning workflows: Recognises the unique challenges of machine learning, especially deep learning, requiring citation of the original method, access to the developed model and validation data, clear identification of pretrained models, and for retrained or new models, making full training and testing data, training metadata, and the code used for training available.

### 2.2 Reproducibility standard

In this work, we focus on the idea of reproducibility as it is used in computational research. Claerbout and Karrenbach^7^ describe reproducibility as the situation where all data and code are available, so another person can run the same analysis again and get the same results. The Association for Computing Machinery (ACM) also uses a similar meaning: an independent group should be able to obtain the same outputs when using the same data and the same software environment. In our study, we follow this definition. We do not try to repeat the experiment with new data or new methods. Instead, we run the workflow with exactly the same dataset and the same software that the authors provided. Our goal is to see if the same results can be reached under these same conditions. For this purpose, we use the QUAREP‐LiMi checklists as a guide to help us evaluate the level of reproducibility in a structured and transparent way.

### 2.3 Reproduction protocol

The Target Paper's published code and data were subjected to a rigorous reproduction attempt across multiple environments to test for environment‐agnostic reliability based on the provided instructions. The key steps were:

1. Local environment setup: An attempt was made to build the environment locally using the documentation provided, revealing essential, unlisted dependencies (e.g., pyyaml, tifffile) and mandatory Python (3.7) and CUDA (10.1) version downgrades.
2. Containerisation test: The search for a functional Dockerfile or robust environment configuration file was conducted to assess adherence to the ‘ideal’ standard of portable, containerised execution.
3. Cloud environment test (Google Colab): An attempt was made to execute the workflow within a common, pre‐configured cloud platform (Google Colab) to specifically assess the barrier to entry for users without high‐end local hardware or complex setup knowledge.
4. Execution and verification: All primary analysis scripts (e.g., ‘run‐functions') were executed. The assessment focused on the success rate, the clarity of diagnostic output, and the ease of verifying that the generated outputs matched the Target Paper's reported results.

## 3 EVALUATION FINDINGS: THE REPRODUCIBILITY GAP

The application of the standardised methodology revealed a significant disparity between the Target Paper's stated commitment to reproducibility and the practical experience of executing the workflow. While the Target Paper satisfies checklist defined ‘minimal’ requirements for data and code availability, the evaluation identified additional challenges in documentation, metadata, and environment specification that affected the practical execution of the workflow.

### 3.1 Data availability and metadata compliance

The authors successfully met the minimal requirement of the Machine Learning Workflow Checklist by providing segmented and tracked data in .NPY files, alongside dedicated example training and testing sets for both synthetic and real data. Code and data were publicly shared on a GitHub repository.

The analysis utilises data stored in .NPY files, specifically a segmentation image (segmentation.npy) and track data (lineage.npy). The segmentation.npy files are described as NumPy arrays with dimensions [T,H,W] for image sequences, where cell borders are set to 0. The lineage.npy files are NumPy arrays containing 10 columns, including Timeframe, CellID, PositionX, and PositionY.

The project provides two sets of examples for synthetic data (data/simulation/del‐div/sample0 for training and sample1 for testing) and two sets for real sample segmentation (data/epidermis/paw/W‐R1 for training and W‐R2 for testing). This provision of validation input and output data, including both training and testing subsets, meets the ‘minimal’ requirement for Machine Learning Workflows and the requirement for example input and output data for New Workflows.

The evaluation identified three compliance issues:

1. Non‐standard format: The reliance on the nonproprietary .NPY format for the image‐derived data does not natively enforce structured microscopy metadata, thus does not adhere to the “ideal” standard of the Image Publication Checklist, which recommends formats like OME‐TIFF to retain rich, structured image metadata.
2. Absence of essential metadata: A significant limitation with respect to current reproducibility expectations was the probable absence of essential metadata, particularly explicit pixel size and timestamps (μm/pixel, imaging intervals). The explicit provision of scale information is a minimal requirement for published image analysis, as its absence may hinder reproducibility of quantitative measurements derived from the images (e.g., velocities, distances). While the text of the paper mentions a region size, and all images have the same size, this information is not embedded in the data.
3. Modern long‐term archival stability: While GitHub is an accessible platform, the lack of an archived repository with a Digital Object Identifier (DOI) (e.g., via Zenodo) falls short of the recommended standards for long‐term data preservation, versioning and citability.

### 3.2 Code quality and workflow execution

The practical execution of the workflow presented notable challenges by code structure and documentation shortcomings, preventing the achievement of the ‘recommended’ and ‘ideal’ standards of the New Workflow Checklist.

1. Library documentation: The README meets the ‘minimal’ requirement by listing precise software versions for *pytorch* (1.6.0), *python* (3.7.7), *dgl* (0.5.0), *numpy* (1.18.5), *matplotlib* (3.3.1), and *scikit‐learn* (0.23.2). Furthermore, the stated use of a Docker container (pytorch:1.6.0‐cuda10.1‐cudnn7‐runtime) aims for the ‘recommended’ to ‘ideal’ standard of providing workflows packaged in software containers to minimise environmental variations.
2. Code structure and navigation: The code is not structured as a standard Python package, and key operations are performed by ‘run‐functions’ nested in subfolders that rely on hardcoded, relative paths. This structure may make navigation and reuse more difficult, isolating specific components, and reusing individual functions.
3. Documentation deficiencies: The code base mostly lacks standard docstrings to explain the function, input, and output of major scripts. Furthermore, while the general sequence of steps is described, the documentation fails to include a detailed discussion of the rationale, assumptions, and limitations for each step/component, a key element of the ‘recommended’ standard. 
4. Verification not fully achievable despite successful run: Crucially, while the code executed to completion without producing runtime errors (a positive finding), the scripts provided very little diagnostic output or clear information regarding the generated results. Multiple files and folders were generated in the indicated results directory, but due to the lack of documentation on the expected file names, format, and content of the final outputs, we were unable to confirm the completeness or scientific fidelity of the results. This low feedback severely reduces the clarity of the final validation step.

### 3.3 Environment setup and barrier to entry

The major challenge to reproducibility from the user perspective was encountered during the environment setup, which demanded substantial, undocumented manual intervention, raising the barrier to entry from a minor hurdle to a complex programming task.

1. Incomplete containerisation support: Although the project intended to meet the ‘ideal’ standard of environmental transparency via containerisation, a functional Dockerfile was missing from the repository, rendering the specific build environment non‐transparent and makes reproducing the exact environment difficult to rebuild reliably. Only name of the base Docker image was specified: pytorch:1.6.0‐cuda10.1‐cudnn7‐runtime. The container did not have the needed packages installed. The Dockerfile for building a custom image with the needed dependencies is missing as well. There is a notable intent to containerisation (positive finding), but without follow‐through.
2. Undocumented libraries: Any setup required manual installation of unwritten dependencies (e.g., *pyyaml* (6.0.1), *tifffile* (2021.11.2)). This dependency management limitation is directly linked to the absence of standard installation files like requirements.txt or setup.py.
3. Cloud environment status: The attempt to run the workflow on a pre‐configured cloud environment (Google Colab) confirmed the difficulties presented by the missing environment files, requiring the same time‐consuming and error‐prone manual setup process, which suggests that the workflow is not robustly portable. While it was possible to run the code after manually downgrading the core Python version and installing specific packages, it would not be considered an entry‐level task for a biologist.

### 3.4 Related tools

The issues of environment setup, metadata adherence, and workflow verification encountered with the Target Paper are not unique, highlighting a widespread need for robust, community‐supported computational solutions in quantitative biology. To guide researchers toward better practices and to highlight existing solutions, we introduce several representative examples from a closely related technical field, cell tracking, where numerous software tools have been developed, some achieving a high level of implementation and reproducibility. First, cell‐tracker‐gnn^8^ is highly relevant to the machine learning pipeline used in this study. It provides a direct implementation of a Graph Neural Network (GNN) for cell tracking in microscopy videos, built on PyTorch Geometric (PyG), a standard library for GNN development. Its open‐source availability and reliance on widely used deep learning frameworks (PyTorch, PyG) ensure high reproducibility of both tracking and graph construction processes. Second, btrack (Bayesian Tracker)^9^, ^10^ is a modern Python‐based tracking library that employs a Bayesian framework for multi‐object tracking. It is particularly effective for reconstructing trajectories in dense live‐cell imaging data and can recover multi‐generational lineage information. As GNN models are typically implemented in Python (e.g., PyTorch or TensorFlow), btrack enables seamless integration into an end‐to‐end, reproducible pipeline. Third, u‐track^11^ is a well‐established MATLAB‐based software and is widely regarded as a gold standard for multiple‐particle tracking. It can handle dense particle fields, close tracking gaps, and capture complex events such as merging and splitting. Due to its widespread use and robustness, it serves as a reliable tool for generating initial cell trajectories, which can subsequently be exported and used in GNN‐based analyses. However, proprietary tools like MATLAB present a licensing barrier to reproducibility. In addition, the Cell Tracking Challenge has played an important role in promoting reproducibility in this field by encouraging the submission of software in standardised formats. These benchmarks provide valuable references for evaluating and selecting reproducible tracking pipelines. Bioimage Informatics Index (BIII) (https://biii.eu/) also provides a curated repository of bioimage analysis tools with standardised descriptions, serving as a useful reference for selecting reproducible methods.

## 4 DISCUSSION AND ACTIONABLE RECOMMENDATIONS

The evaluation confirms that while the Target Paper contributes a novel Graph Neural Network approach to a complex problem in quantitative biology, its utility for the wider scientific community is limited by challenges related to practical reproducibility. Importantly, this analysis reflects a retrospective evaluation of a study published in 2022, conducted using reproducibility standards and checklists that were formalised only later. The core issue is the systemic difficulty to translate the discussed and explained content of the paper into a functional, user‐friendly, and portable computational workflow. This section outlines the broader implications of these findings for the quantitative biology community and provides a dedicated, prioritised action plan for the original authors.

### 4.1 Broader implications for quantitative biology

The findings regarding the Target Paper can serve for the community as a critical case study illustrating common pitfalls in publishing high‐end computational biology:

- The practical re‐execution challenge: The paper's public release of code and data created an appearance of reproducibility that can mask underlying challenges. Our experience demonstrates that compliance even with current ‘minimal’ requirements (e.g., uploading files) is insufficient. Achieving robust reproducibility typically requires satisfying ‘recommended’ and ‘ideal’ standards for code quality, environment management, and comprehensive documentation. The critical challenge was that execution success without verification. The provided documentation is difficult to interpret without verification steps. Code running without error only confirms script syntax; the absence of documented, expected outputs prevents the crucial final step of confirming scientific fidelity. The lesson from this observation is the need to improve how execution outcomes and output verification are handled and documented, so that the validity of the results can be assessed in an accessible and transparent manner.
- Barriers to adoption and systematic cost: The need for manual dependency installation and the absence of containerisation create a significant barrier to entry. This not only prevents replication but also limits the adoption of the methodology by non‐specialist labs or researchers attempting to apply the GNN framework to different datasets. This inefficiency is not isolated; a 2016 Nature survey found that over 70% of scientists struggle to reproduce the experiments of others,^12^ leading to an estimated annual cost of billions of dollars in lost efficiency in preclinical research alone.^13^
- The role of checklists: This evaluation highlights the crucial role of community‐developed standards (like the checklists used here) in moving beyond general guidelines. They provide a structured framework to objectively measure the quality of a scientific release, fostering a culture where reproducibility is an engineered feature rather than an assumed by‐product.

### 4.2 Proposed remediation plan

This plan provides a structured, prioritised list of actions necessary for the authors of the Target Paper to transition their project from current codebase to a scientifically robust, community‐ready resource.

#### 4.2.1 Immediate/minimal requirement fixes

These issues violate basic standards and must be addressed immediately to ensure data integrity and basic execution:

1. Metadata correction: Add explicit, unambiguous metadata to the repository documentation, specifically documenting the pixel size, scale bar information, and imaging intervals (timestamps) used for the original dataset.
2. Dependency management: Create and test a standard, machine‐readable dependency list, such as a requirements.txt or an environment.yml file, to eliminate the need for manual, trial‐and‐error dependency installation and system downgrades.
3. Data preservation: Move the core dataset files from the GitHub repository to a dedicated, public data repository (e.g., BioImageArchive or Zenodo) and obtain a Digital Object Identifier (DOI) for stable, long‐term archival and citation. Ideal approach would be to have separate repository for code and for the image data.

#### 4.2.2 Recommended/workflow improvement fixes

These actions address code quality and documentation issues that currently prevent easy use and adaptation:

1. Code packaging: Refactor the codebase into a standard Python package structure. This will eliminate the reliance on hardcoded paths and nested ‘run‐functions’, making individual modules importable and reusable.
2. Inline documentation: Implement comprehensive docstrings for all major functions and scripts, clearly detailing inputs, outputs, data types, and any necessary preconditions or side effects.
3. Verification documentation: Create a simple, step‐by‐step tutorial, preferably in a Jupyter Notebook or detailed README, that includes small sample data and illustrates the entire workflow. This documentation must explicitly detail the expected final output structure, including the names, locations, and contents of the files and folders generated, which is necessary to confirm that the execution was scientifically successful.

#### 4.2.3 Ideal/portability and transparency fixes

These are advanced steps required to meet the highest standards of transparency and environment portability:

1. Container provisioning: Develop and test a functional Dockerfile (or Singularity/Apptainer container) that successfully builds the complete environment without external manual intervention (including all the correct Python Libraries and CUDA versions), ensuring the workflow can be executed identically by any user.
2. Comprehensive rationale: Augment the documentation to include a detailed rationale, assumptions, and limitations for each step of the workflow, particularly regarding segmentation, graph construction, and the specific choices made for the GNN architecture.

## 5 CONCLUSION

The systematic assessment of the Target Paper's GNN workflow highlights a crucial, solvable challenge: transitioning from open data principles to achieved computational reproducibility. As pioneers defined it, the goal is to allow a colleague to ‘run the same software on the same input data and obtain the same results’.^14^ The challenges encountered—such as limited metadata or incomplete environment specification—highlight a lack of a defined environment, and a critical absence of verification documentation—underscoring the importance of treating reproducibility as an engineered component of the publication process.

It is worth emphasising that the authors demonstrated a clear commitment to reproducibility, even though they did not yet have community checklists or formal standards to guide them. Sharing the complete dataset, providing executable scripts, releasing both synthetic and biological training and testing sets, and making their GNN architecture transparent were substantial contributions at the time. These actions reflect genuine openness and a willingness to support reuse—values that have since become central pillars of reproducible computational biology. Their work helped shape the evolving expectations that modern checklists now codify.

As Miura and Nørrelykke^15^ argue in their work on reproducible image analysis, establishing a new norm requires a ‘precise description of the image processing and analysis applied’. This necessity demands a unified commitment to improvement (Figure 2) across all stakeholders:

- For authors: The immediate priority is to adopt DevOps practices and literate programming. By utilising tools like BIAFLOWS,^16^ which offers a versatile and extensible integrated framework for workflows, authors can easily meet the ‘Ideal’ standard of environment portability and verification.
- For reviewers and journals: We must mandate the use of structured, community‐developed checklists to verify computational rigor before publication, thereby moving beyond checking for mere data upload.
- For the community: This case is not a critique of individuals, but a call to collective action. By mandating standards like FAIR data principles and rewarding high‐quality environment specifications and verification documentation, we can eliminate the costly ‘forensic research’ currently needed to validate published work, ultimately accelerating scientific discovery in quantitative and computational biology.

**FIGURE 2:**
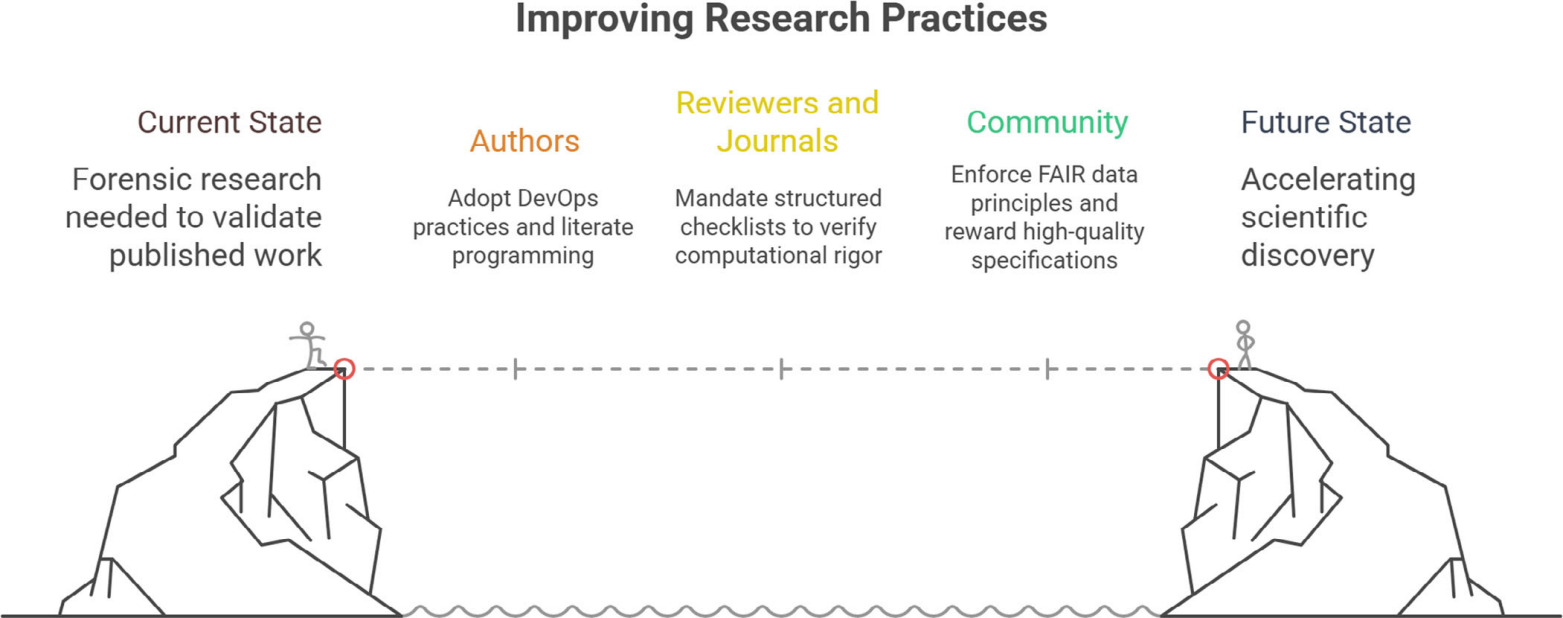
Unified approach to improve research practices.

## CONFLICT OF INTEREST STATEMENT

K.S. is employed part‐time by LPIXEL Inc.

## DISCLOSURE OF AI USE

Artificial intelligence tools were used in the preparation of this manuscript as follows: Google NotebookLM was employed to export, organise and summarise various types of research notes. Grammarly Premium was used to improve grammar and style. All AI‐assisted outputs were reviewed and verified by the authors to ensure accuracy, originality, and compliance with Wiley's ethical and editorial standards. No AI tools were used to generate, alter, or manipulate original research data or results.

## Data Availability

The data that support the findings of this study are openly available in Zenodo at https://doi.org/10.5281/zenodo.17777052, reference number,^17^ under Apache License 2.0 licence. We acknowledge that the author list on the Zenodo repository differs from the manuscript authors; the Zenodo metadata reflects the technical maintainers of the archived datasets. The checklists, reference number,^6^ can be downloaded as printable files from https://doi.org/10.5281/zenodo.7642559. A completed checklist applied to the target paper is provided as supplementary material. The companion Jupyter Book can be found at https://quarep‐limi.github.io/WG12_checklists_for_image_publishing/intro.html.

## Footnotes

1 Global BioImage Analysts' Society (GloBIAS) home page: https://www.globias.org/

2 The checklists^6^ can be downloaded as printable files from https://doi.org/10.5281/zenodo.7642559. The companion Jupyter Book can be found at https://quarep‐limi.github.io/WG12_checklists_for_image_publishing/intro.html
